# Multifaceted role of geminivirus associated betasatellite in pathogenesis

**DOI:** 10.1111/mpp.12800

**Published:** 2019-06-18

**Authors:** Prabu Gnanasekaran, Reddy KishoreKumar, Dhriti Bhattacharyya, R. Vinoth Kumar, Supriya Chakraborty

**Affiliations:** ^1^ Molecular Virology Laboratory, School of Life Sciences Jawaharlal Nehru University New Delhi 110 067 India

**Keywords:** βC1, betasatellites, chloroplast, defence, Geminivirus, interaction, pathogenesis

## Abstract

Begomoviruses have emerged as a group of plant pathogens that cause devastating diseases in a wide range of crops in tropical and subtropical regions of the world. Betasatellites, the circular single‐stranded DNA molecules with the size of almost half of that of the associated helper begomoviruses, are often essential for the production of typical disease symptoms in several virus‐host systems. Association of betasatellites with begomoviruses results in more severe symptoms in the plants and affects the yield of numerous crops leading to huge agroeconomic losses. βC1, the only protein encoded by betasatellites, plays a multifaceted role in the successful establishment of infection. This protein counteracts the innate defence mechanisms of the host, like RNA silencing, ubiquitin‐proteasome system and defence responsive hormones. In the last two decades, the molecular aspect of betasatellite pathogenesis has attracted much attention from the researchers worldwide, and reports have shown that βC1 protein aggravates the helper begomovirus disease complex by modulating specific host factors. This review discusses the molecular aspects of the pathogenesis of betasatellites, including various βC1‐host factor interactions and their effects on the suppression of defence responses of the plants.

## Introduction

Specific interactions between the virus and the host proteins are prerequisites for both the pathogenicity determinant to execute its virulence function and the plant to activate its anti‐virus surveillance mechanisms (Kong *et al*., 2014). But the complexities of such interactions impose challenges in understanding the detailed mechanisms of the defence responses of the plant generated against the invading viruses. The defence responses in plants are manifested through the pathways like R‐gene‐mediated defence, RNA silencing, ubiquitin‐mediated proteasomal degradation etc. and involve several host factors that adversely regulate virus accumulation (Bhat *et al*., 2013; Bhattacharyya *et al*., 2015; Eini *et al*., 2009b; Li *et al*., 2014a; de Ronde *et al*., 2014; Shen *et al*., 2016).

The plant hormones such as salicylic acid (SA) and jasmonic acid (JA) contribute to the defence responses by eliciting the expressions of specific hormone‐responsive genes. These gene products generate an antiviral state that restricts the invading pathogens (Spoel *et al*., 2007). During both compatible and non‐compatible host‐pathogen interactions, plants undergo autophagy (Alazem and Lin, 2015), which as an innate immunity response, degrades the viral protein(s) and reduces the viral infection (Haxim *et al*., 2017). In addition, necrotrophic pathogens, biotrophic pathogens and plant viruses induce the production of defence‐related reactive oxygen species (ROS), which could also activate autophagy. Furthermore, chloroplasts, as the sites for production of defence hormones, play a central role in innate immunity of the plants and aid in restricting the viral spread and systemic infection (Bhattacharyya and Chakraborty, 2018). Furthermore, the photosystem‐II dependent defence‐related ROS production also could induce autophagy and programmed cell death (Doyle *et al*., 2010).

Geminiviruses severely interfere with the physiology of the host plants and are responsible for major crop losses in economically important dicots or monocots globally (Navas‐Castillo *et al*., 2011). These viruses belong to a family of small, non‐enveloped, single‐stranded DNA viruses possessing a circular genome ranging in size from 2.5 kb to 3.2 kb (in case of monopartite viruses) and from 4.8 kb to 5.6 kb (in case of bipartite viruses), and are encapsidated in particles consisting of two joined incomplete icosahedra (Navas‐Castillo *et al*., 2011; Zerbini *et al*., 2017). The International Committee on Taxonomy of Viruses classified the family *Geminiviridae* into nine different genera, namely, *Becurtovirus, Begomovirus, Capulavirus, Curtovirus, Eragrovirus, Grablovirus, Mastrevirus, Topocuvirus *and *Turncurtovirus *(Zerbini *et al*., 2017). The genus *Begomovirus* consists of ~ 350 species that are geographically widespread and transmitted by the whitefly, *Bemisia tabaci*.

Monopartite begomoviruses contain a single genome of size approximately 2.7 kb, named as DNA‐A. The genomes of bipartite begomoviruses consist of two genomic components namely, DNA‐A and DNA‐B (Zerbini *et al*., 2017). DNA‐A genome encodes for coat protein (CP, AV1/V1), pre‐coat protein (AV2/V2; absent in the New World bipartite begomovirus), replication associated protein (Rep, AC1/C1), transcriptional activator protein (TrAP, AC2/C2), replication enhancer protein (REn, AC3/C3), and C4 protein (AC4/C4). DNA‐B genome encodes for nuclear shuttle protein (NSP, BV1) and movement protein (MP, BC1) (Rojas *et al*., 2005; Zerbini *et al*., 2017). Studies in the last few decades revealed the association of begomoviruses with molecules like betasatellite, alphasatellite and deltasatellite (Kumar *et al*., 2015; Mansoor *et al*., 2006; Nawaz‐ul‐Rehman and Fauquet, 2009; Saunders *et al*., 2000; Vinoth Kumar *et al*., 2017). Satellites are extra viral components that have genomes of almost half or less than half of the size of that of the helper viruses and do not encode CP. But these satellite molecules can influence pathogenesis and accumulation of the associated helper viruses and are considered as part of the begomovirus disease complex (Gnanasekaran and Chakraborty, 2018).

Alphasatellites are autonomously replicating satellite‐like molecules with a circular single‐stranded DNA molecule of the size 1.3 kb to 1.4 kb and they need helper viruses for their movement inside the host plant and for vector transmission. The self‐replication of alphasatellites inside the host plant is accomplished by the alpha‐Rep protein (approximately of 37 kDa) encoded by the single open reading frame (ORF) present in its genome. This alpha‐Rep binds to the helper virus‐encoded Rep protein (Nawaz‐Ul‐Rehman *et al*., 2010). Interestingly, the interaction between alpha‐Rep and helper virus Rep leads to inhibition of accumulation of betasatellites, which too, rely on helper virus Rep for their replication (Nawaz‐Ul‐Rehman *et al*., 2010; Vinoth Kumar *et al*., 2017). Deltasatellites are non‐coding, circular single‐stranded satellite DNA molecules associated with begomoviruses (Lozano *et al*., 2016) and are reported to reduce both the accumulation of viral DNA and helper virus‐mediated symptom development in the infected plants (Ceniceros‐Ojeda *et al*., 2016; Hassan *et al*., 2016).

Betasatellites are circular single‐stranded DNA molecules of approximately 1.3 kb, which are associated with begomoviruses and are often found to be necessary for symptom development as well as increased accumulation of viral nucleic acids in the host (Briddon *et al*., 2001; Jose and Usha, 2003; Saunders *et al*., 2000). Betasatellites share little sequence similarity with their helper viruses and are completely dependent on the helper viruses for replication, encapsidation, movement and insect transmission (Briddon *et al*., 2008; Hanley‐Bowdoin *et al*., 2013).

The associations of betasatellites with the majority of the monopartite begomoviruses and their promiscuous trans‐replication by diverse helper begomoviruses have made them a serious threat to the agro‐economy. Reports have suggested that βC1 protein, the only protein encoded by the betasatellite genome, elevates the disease manifestation by suppressing the plant defence machinery and by augmenting the accumulation of the helper begomovirus disease complex (Bhattacharyya *et al*., 2015; Li *et al*., 2014b; Mansoor *et al*., 1999; Zhong *et al*., 2017). Although the aspects like the evolution of betasatellites have been reviewed previously (Zhou, 2013; Nawaz‐ul‐Rehman and Fauquet, 2009), there is no recent reviews on the role of βC1 in disease development. The current review provides an exhaustive account of betasatellite pathogenicity with an emphasis on the molecular aspects of different host‐virus interactions. It starts with a brief overview of the genomic organization and the genetic diversity of betasatellites followed by the discussion on the role of betasatellites as an important part of the begomovirus disease complex. Next, various interactions between βC1 and host factors as well as the implications of those interactions on the suppression of plant defence mechanisms and disease development are presented.

## Genomic Organization and Replication of Betasatellites

The sequence analysis of different betasatellites associated with various begomoviruses revealed the presence of three common structural features: (i) an A‐rich region, (ii) a 150–200 nucleotides long satellite conserved region (SCR) containing a potential hairpin loop structure with nonanucleotide TAATATTAC, (iii) a single ORF encoding multifunctional protein βC1, of size approximately 13 kDa–14 kDa, in its complementary sense‐strand. In all known functional betasatellites, the location of this ORF is conserved with the start codon placed between 544 nt and 570 nt, and the stop codon placed between 195 nt and 209 nt from the origin of replication (Briddon *et al*., 2003; Mansoor *et al*., 2003a). The replication of a betasatellite is mediated by the replication associated protein (Zerbini *et al*., 2017) encoded by the helper begomovirus. Betasatellites undergo a rather relaxed interaction with the helper viruses, as many of them are trans‐replicated by various helper viruses (Dry *et al*., 1997; Mansoor *et al*., 2003a; Ranjan *et al*., 2014; Saunders *et al*., 2002). Although betasatellites depend on the Rep proteins of the helper viruses for replication, they usually do not possess the conserved iterons in their genome (Rep binding sequences) like the helper viruses. Through deletion analysis, the origin of replication of betasatellites was identified to be encompassing the SCR, a part of the intergenic region upstream of the SCR, and the ubiquitous nonanucleotide/stem‐loop structure (Saunders *et al*., 2008). A study with *Tomato yellow leaf curl China virus* (TYLCCNV), *Tobacco curly shoot virus *(TbCSV), and their respective betasatellites namely, *Tomato yellow leaf curl China betasatellite* (TYLCCNB) and *Tobacco curly shoot betasatellite* (TbCSB) has shown the presence of a novel Rep binding motif (RBM) in the genome of betasatellites. The different binding affinities of RBM with cognate and non‐cognate Rep proteins correlate with promiscuous selection and efficiency of trans‐replication of various betasatellites (Zhang *et al*., 2015). The role of host‐specific adaptability in the trans‐replication and maintenance of betasatellites by various begomoviruses was also demonstrated (Ranjan *et al*., 2014).

## Genetic Diversity of Betasatellites

Symptom severity caused by the viruses is prompted by their evolutionary fitness, which in turn depends on the internal genetic modifications achieved primarily by mutation, recombination and pseudo‐recombination (reassortment) (Seal *et al*., 2006). In addition, external factors such as climatic changes, synergistic/antagonistic effects of the associated helper viruses and the specific mediating vectors, play roles in the evolution of viruses (Acosta‐Leal *et al*., 2011). Amongst others geminiviruses showed enormous genetic variations from their proposed prokaryotic plasmid origin (Krupovic *et al*., 2009). Betasatellites rapidly adapt to diverse geminiviral components leading to the generation of unique disease complexes and expanding their ecological niches by increasing the host range, disease severity and enhancement of the vector performance (Mansoor *et al*., 2006; Nawaz‐ul‐Rehman and Fauquet, 2009; Patil and Fauquet, 2010; Sattar *et al*., 2017; Zubair *et al*., 2017).

**Figure 1:**
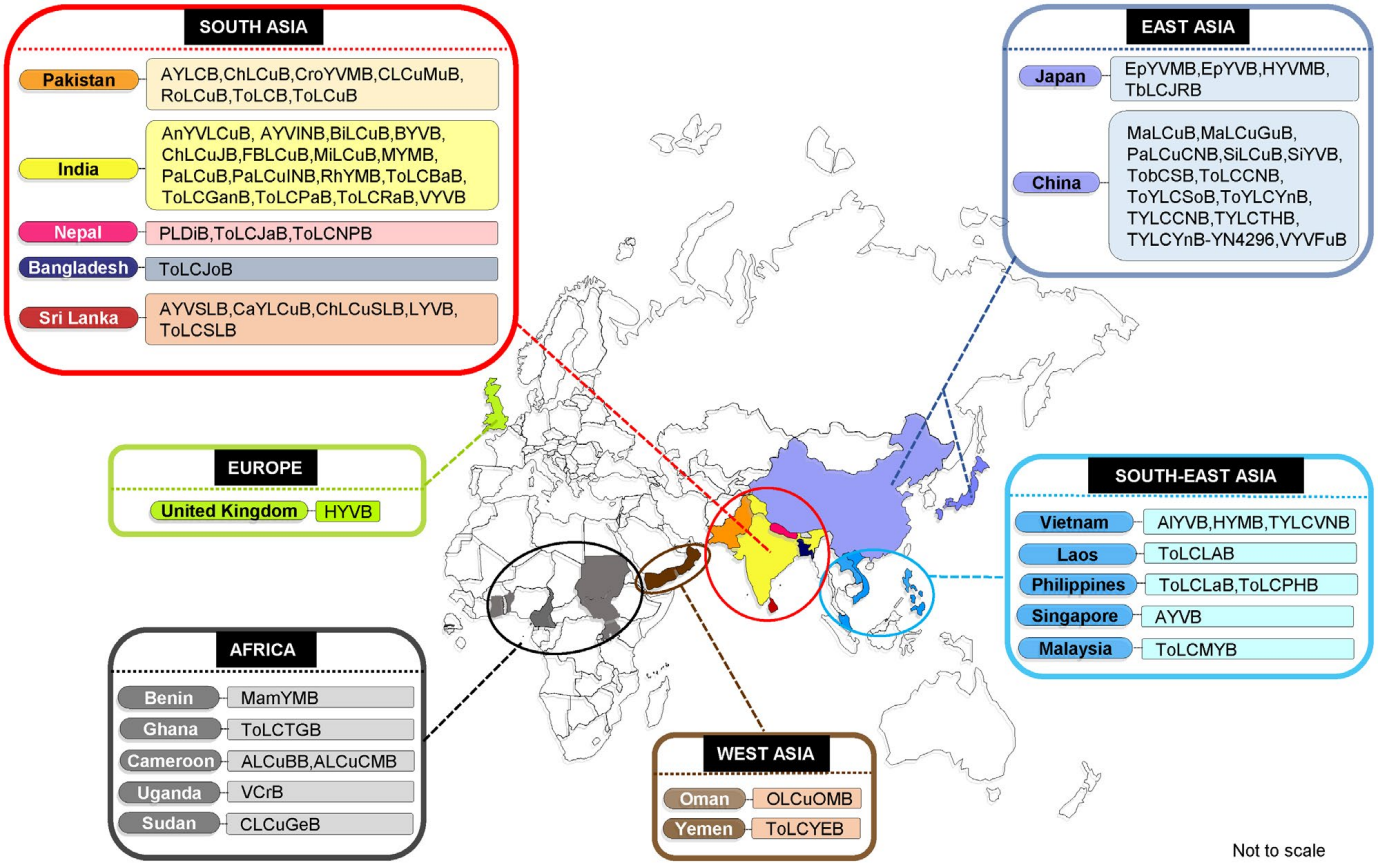
Geographical distribution of distinct betasatellites across the ‘Old World’ countries. The presence of distinct betasatellites identified from different geographical locations is indicated in multiple colours. The full name of betasatellites mentioned here is provided in Table. S1.

At least 66 distinct betasatellites, associated with diverse helper viruses and numerous disease complexes that infect a vast range of hosts, identified from around 20 countries in the Asian, African and European continents belonging to the “Old World” (Table. S1). The majority of these molecules (59/66) was identified from Asia, and amongst these molecules 32 distinct betasatellites were reported from the Indian subcontinent alone (Bangladesh, India, Nepal, Pakistan and Sri Lanka) (Fig. 1). In addition, high genetic diversity amongst betasatellites was found in China, too. Furthermore, the isolates of *Cotton leaf curl* *Gezira betasatellite *(CLCuGeB) and *Ageratum leaf curl Cameroon betasatellite* are the predominant betasatellite groups reported from the West and Central regions of Africa, while tomato leaf curl associated betasatellites are prevalent in Oman (Khan *et al*., 2014; Leke *et al*., 2015). Many of these satellites were isolated from the plants belonging to the families Solanaceae, Asteraceae or Malvaceae (Table. S1).

Betasatellites associated with chilli leaf curl disease in India, for instance, *Tomato leaf curl Bangladesh betasatellite,* contain high nucleotide variability and a high nucleotide substitution rate in the βC1 coding regions (Kumar *et al*., 2015). The genetic variation of begomovirus populations is mainly attributed to the mutational dynamics involving point mutations generated by nucleotide substitution (Lima *et al*., 2017). As begomoviruses use the host DNA polymerase, the replication fidelity of these viruses likely should be the same as that of the host. Therefore, the high mutation rate might be due to a less stringent repair process of the geminivirus genomes that lack the proper methylation patterns for the host exonucleases (Sanz *et al*., 1999). In the case of the correctly methylated begomovirus genomes, base‐excision repair might not take place as the genomes are only transiently double‐stranded during rolling‐circle replication. Nevertheless, the possibility of the viruses recruiting a more error‐prone polymerase from the host's nucleus for their own replication has not been ruled out (Duffy and Holmes, 2008). Additionally, betasatellites facilitate the conditions that favour the whitefly vectors and thus, promote the propagation of the disease complexes. These conditions include induction of the positive behavioural responses through enhanced linalool emission in plants, suppression of the host's anti‐herbivory responses and increasing the fecundity of the vectors (Jia *et al*., 2012; Jiu *et al*., 2007; Li *et al*., 2014b; Salvaudon *et al*., 2013).

Mixed infection is a source of recombination and pseudo‐recombination that contribute to begomovirus‐betasatellite diversity. Amongst chilli‐infecting betasatellites, the A‐rich region and SCR have been reported as hot spots for recombination (Kumar *et al*., 2015). Reassortment between CLCuGeB and *Tomato yellow leaf curl Mali virus* caused more severe growth stunting and deformation of leaves than the usual leaf curling phenotypes in tomato (Chen *et al*., 2009).

## Association of Betasatellite With Disease Complex Influences the Begomovirus Pathogenesis

The earliest account of a plant virus disease, manifested by the yellow vein symptom in eupatorium plants, was found more than a millennium ago in Japanese literature. The causative agent of the disease was later identified as a begomovirus‐betasatellite disease complex (Saunders *et al*., 2003). Although initially attributed to *Ageratum yellow vein virus* (AYVV), a monopartite begomovirus (Tan *et al*., 1995), the typical yellow vein symptoms of *Ageratum conyzoides* infected by the begomovirus (Swanson *et al*., 1993) was eventually hypothesized to be associated with the presence of additional factors (Saunders *et al*., 2000; Saunders and Stanley, 1999). Subsequently, a complete betasatellite molecule was isolated from the diseased Ageratum plant (Saunders *et al*., 2000). Several studies reported the indispensable role of betasatellite in the establishment and maintenance of diseases in the host plants (Briddon *et al*., 2001; Jose and Usha, 2003; Kumari *et al*., 2010; Saunders *et al*., 2000; Singh *et al*., 2012).

Phylogenetic analyses of the viral genomes showed that betasatellites have undergone co‐evolution with their helper viruses (Zhou *et al*., 2003). Betasatellites are widespread amongst the ‘Old World’ begomoviruses that are mostly monopartite in nature (Kumar *et al*., 2015; Saeed *et al*., 2007; Zubair *et al*., 2017). In addition, betasatellites have also been reported to be associated with a few bipartite begomoviruses such as *Sri Lankan cassava mosaic virus*, *Tomato leaf curl Gujarat virus* and *Tomato leaf curl New Delhi Virus* (ToLCNDV) (Jyothsna *et al*., 2013; Ranjan *et al*., 2014; Sivalingam and Varma, 2012). This promiscuity is particularly threatening for the agronomy as, even in the absence of any helper begomovirus, a betasatellite can be maintained by a mastrevirus in the field‐grown wheat plants elevating the accumulation of the helper virus (Kumar *et al*., 2014). Such associations of betasatellites with the viruses of different genera might generate severe disease complexes that may invade new economically important crops. Furthermore, the synergistic interaction between the betasatellite and multiple helper viruses enhanced the viral DNA replication in resistant chilli cultivar and might result in breakdown of the natural resistance (Singh *et al*., 2016).

## The Contribution of the A‐Rich Region and SCR in Betasatellite‐Mediated Disease Development

As mentioned earlier, apart from the ORF of the pathogenicity determinant βC1, the biologically active betasatellite molecules contain two other conserved features—an A‐rich region and an SCR (Briddon *et al*., 2003). The conserved A‐rich region, present even in the naturally occurring but the truncated betasatellites, is probably a ‘stuffer’ sequence essential in maintaining the size of the betasatellite genome (Briddon *et al*., 2003; Mansoor *et al*., 2003b). Although replication and encapsidation were not affected in the betasatellites with deleted A‐rich regions, such mutant betasatellites induced milder symptoms on *Nicotiana benthamiana*. This region includes the putative enhancer elements for the βC1 promoter and hence might regulate the symptom severity by affecting the protein expression (Tao *et al*., 2004). The promoter of TYLCCNB is phloem‐specific and capable of inducing vein thickening in the host (Guan and Zhou, 2006), while the promoter of TbCSB, which is constitutively expressed, is unable to induce similar symptoms. The study of Ding *et al*. (2009) involved different hybrid molecules containing the promoters and βC1‐ORFs of TYLCCNB and TbCSB. The result of this study revealed that the promoter of βC1 indeed influences the symptom development. Also, the study of Guan and Zhou (2006) suggested that the vein swelling and enation symptoms induced by TYLCCNV‐DNA infection in the tobacco plants might be due to the phloem‐specific expression of the βC1 gene. These reports indicate that although the pathogenicity factor βC1 protein is the symptom determinant, the promoter of the gene also influences symptom production, at least in the cases of some of the betasatellites. The regulatory element of the viral promoter might interact with distinct host factors (Yin and Beachy, 1995) causing tissue‐specific expression of the gene. Also, the genetic elements in the viral promoter are exposed to the post‐transcriptional gene silencing (PTGS) machinery and the methylation‐mediated suppression of the plant (Dogar, 2006). The promoter of βC1 of the betasatellite associated with cotton leaf curl disease contains G‐box motifs, which is important for the activity of the satellite molecule. This G‐box element is capable of binding to the host factors and is important for the replication of the satellite, too (Eini *et al*., 2009a). Further studies are required to explore the possible role of the non‐coding region, including A‐rich region and SCR, of the betasatellites in interacting with the host factors and the effect of such interactions in pathogenesis.

## The Role of the βC1 Protein, a Pathogenicity Factor, in Disease Development

**Figure 2:**
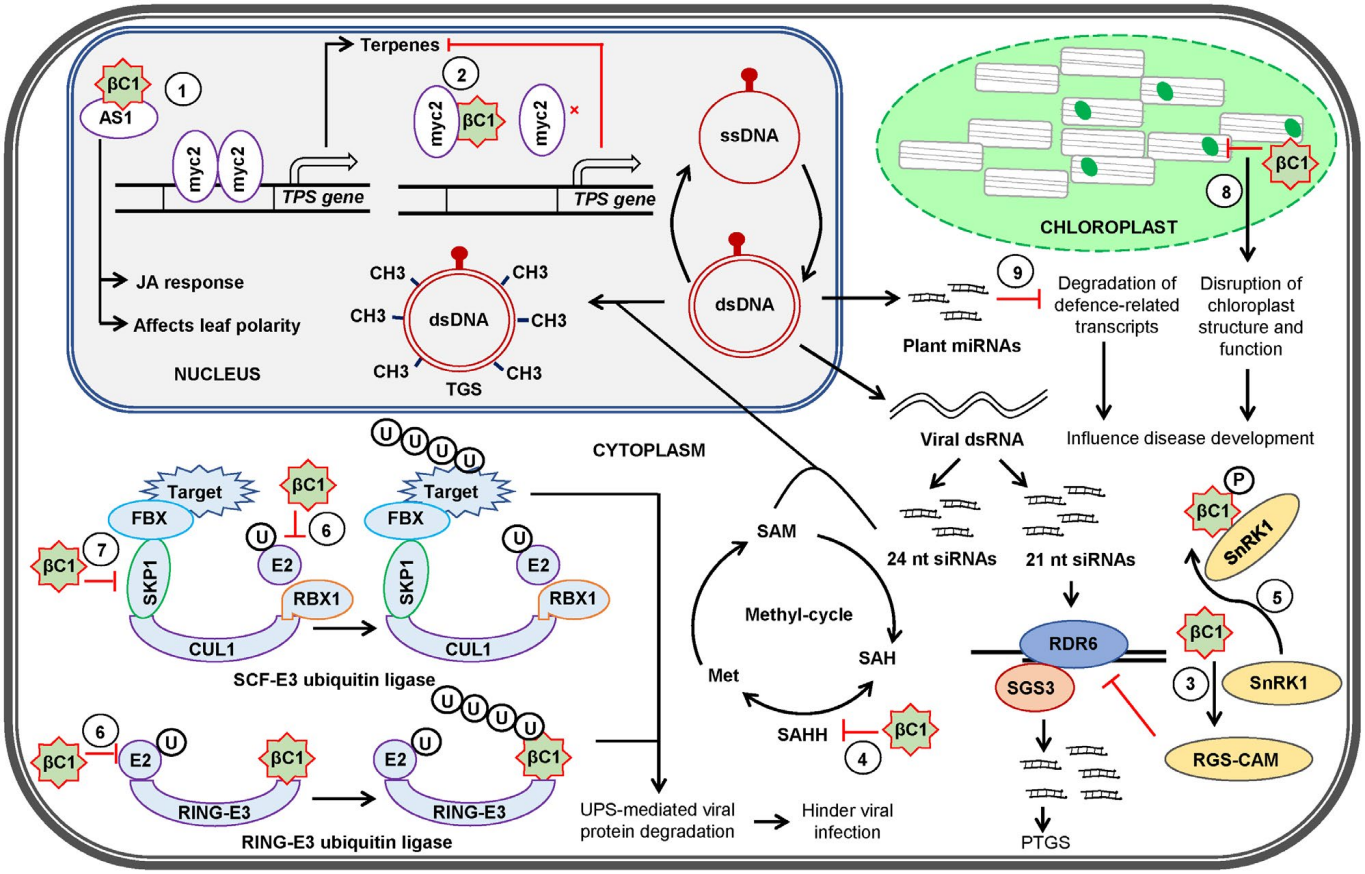
Multifaceted roles of βC1 in the scope of viral pathogenesis. A schematic model depicting the roles of pathogenicity determinant, βC1 in successful pathogenesis. In the host cell, βC1 interacts with ASYMMETRIC LEAVES 1 (AS1) in the molecular disguise of ASYMMETRIC LEAVES 2 (AS2), thereby affecting the jasmonic acid (JA) response and the leaf polarity leading to symptom development (1). The interaction of βC1 with MYC2 diminishes the dimerization of MYC2 and subsequent downstream expression of terpene biosynthesis genes is hampered (2). The βC1 protein‐mediated inhibition of syntheses of JA and terpenes attributes to its influences on virus‐host‐vector tripartite interaction. βC1 acts as a strong suppressor of RNA silencing and affects both transcriptional and post‐transcriptional gene silencing (TGS and PTGS) machinery of the host. βC1 suppresses PTGS by inducing the host rgs‐CAM expression and repressing RDR6 (3). βC1 suppresses TGS by interacting with S‐adenosyl homocysteine hydrolase (SAHH), a methyl cycle enzyme. βC1 inhibits SAHH enzyme activity and eventually impedes the production of S‐adenosyl homocysteine (SAM), an active methyl group donor for methylation (4). As a defence mechanism, host SnRK1 protein phosphorylates βC1, thereby inactivating its ability to act as a suppressor of TGS and PTGS (5). The host RING‐E3 ubiquitin‐ligase (RFP1) polyubiquitinates and degrade the βC1 protein through the ubiquitin/26S proteasome system. βC1 interferes with host ubiquitin‐proteasome pathway via interaction with ubiquitin conjugase E2 (6). βC1 interacts with SKP1 inhibiting SKP1‐CUL1 interaction, and obstructs the SCF‐E3 ligase complex formation, and subsequently preventing the UPS‐mediated viral protein degradation (7). The chloroplast‐mediated defence response is hampered as the ultrastructure and function of the organelle are damaged. The expression of various important host genes including those involved in photosynthesis and defence are impeded by βC1 (8). Differential regulation of diverse set miRNAs that are responsive to betasatellite infection contributes to disease symptom development (9). The various strategies adopted by βC1 to counter the plant defence correlate to its role in pathogenicity determination, the establishment of disease and the symptom development. CH3 indicates methylation, (P) indicated phosphorylation, and (U) indicates ubiquitination.

Studies in the last two decades have established that βC1 acts as the pathogenicity determinant protein during begomovirus pathogenesis (Zhou, 2013). The multitasking βC1 protein suppresses the host defence responses mediated by PTGS, transcriptional gene slicing (TGS), ubiquitin‐proteasome system and defence hormones of the plants (Bhattacharyya *et al*., 2015; Jia *et al*., 2016; Yang *et al*., 2008; Zhou, 2013) (Fig. 2). In an infected plant cell, βC1 protein performs its pathogenicity function by modulating the cellular niche through specifically interacting or targeting the host factors (Table. 1). In addition, βC1 protein contributes to the disease and symptom development by assisting the intracellular and systemic transport of the virus and facilitating the virus‐vector‐host tripartite interactions. The next few sections discuss the prominent molecular aspects of βC1‐mediated pathogenicity.

**Table 1:** βC1‐host protein interactions.

Associated betasatellite	βC1 targeting host factor	Implications in the scope of defence/counter‐defene response	References
*Bhendi yellow vein mosaic betasatellite*	Karyopherin‐α	BYVMV‐movement protein interacts with BYVMB‐βC1, which in turn interacts with karyophilin‐α Putatively facilitates viral movement through the nuclear membrane	Kumar et al., 2006
*Tomato yellow leaf curl China betasatellite*	Asymmetric leaves 1 (AS1)	Suppresses JA‐biosynthesis and JA‐responsive genes Alters MIR165/166 and *HDZIPIII* transcripts	Yang et al., 2008
*Cotton leaf curl Multan betasatellite*	Ubiquitin‐conjugating enzyme (E2)	βC1 modulates the host ubiquitin/26S proteasome pathway by inhibiting the ubiquitin conjugase E2 Reduces polyubiquitination of protein and prevent degradation of the viral protein	Eini et al., 2009b
*Tomato yellow leaf curl China betasatellite*	Sucrose‐nonfermenting1‐related kinase (SnRK1 )	SnRK1 interacts with and phosphorylates βC1 through its kinase domain Phosphorylation inhibits PTGS and TGS suppressor activity of βC1 Attenuates disease symptom and lowers accumulation of viral DNA	Shen et al., 2011; Zhong et al., 2017
*Tomato yellow leaf curl China betasatellite*	S‐adenosyl homocysteine hydrolase (SAHH )	βC1 inhibits the activity of SAHH required for the production of S‐adenosyl methionine, active methyl group donor for methylation reaction Hampers methyl cycle and reduces host and viral genome methylation level	Yang et al., 2011b
*Tomato yellow leaf curl China betasatellite*	Calmodulin‐like protein (rgs‐CaM)	βC1 induces expression of rgs‐CAM, an endogenous regulator of RNA gene silencing Induced level of rgs‐CAM suppresses the host RNA silencing by repressing the expression of RDR6	Li et al., 2014a
*Tomato yellow leaf curl China betasatellite*	The basic helix‐loop‐helix transcription factor (MYC2)	βC1 protein interacts with MYC2, interferes with its dimerization required for binding to the promoter Suppress the terpene biosynthesis genes and establishes virus‐insect vector mutualism	Li et al., 2014b
*Radish leaf curl betasatellite*	Oxygen‐evolving complex (OEC) of PSII	βC1 protein localizes into the chloroplast of the infected plant cell Interferes with the electron transport in PSII probably affecting the OEC of PSII βC1 protein inhibits the chloroplast defence response by affecting the structure and function of the chloroplast	Bhattacharyya et al., 2015
*Tomato yellow leaf curl China betasatellite*	RING‐finger protein (RFP1)	Host ubiquitin‐ligase E3, RFP1 interacts with and polyubiquitinates βC1 and direct them to 26S proteasome‐mediated degradation Delays establishment of geminivirus infection	Shen et al., 2016
*Cotton leaf curl Multan betasatellite*	S‐phase kinase‐associated protein (SKP1)	βC1 protein interacts with SKP1 and interferes with SKP1‐CUL1 interaction and thereby prevents SCF‐E3 ligase complex formation The βC1‐SKP1 interaction impairs SCF^col1^ and subverts JA‐mediated suppression of viral infection cycle	Jia et al., 2016
*Cotton leaf curl Multan betasatellite*	Autophagy protein (ATG8)	Plant ATG8 protein interacts with virulence protein βC1 protein and subsequently induces autophagy Autophagy contributes to the plant innate immunity by degradation of viral protein and restricting its spread	Haxim et al., 2017
*Cotton leaf curl Multan betasatellite*	Argonaute‐1 (AGO1)	βC1 protein physically interacts with AGO1 and possibly targets RNA silencing	Eini, 2017

### RNA silencing

Host plants use the RNA silencing mechanism as an effective antiviral defence strategy, whereas viruses encode silencing suppressor proteins to counter this mechanism (Pumplin and Voinnet, 2013). For instance, a diverse set of novel and defence responsive miRNAs were found to be differentially regulated upon begomovirus‐betasatellite infection (Xiao *et al*., 2014). Various betasatellites were also reported to modulate the accumulation of virus‐derived sRNAs, thereby targeting several stress‐related defence proteins and transcription factors like myeloblastoses (MYBs), and subsequently promoting the virus infections (Wang *et al*., 2016; Yang *et al*., 2011a). This betasatellite‐dependent differential expression of small RNAs could be due to the induction of RNA silencing components such as AGO1 and DCL1, and/or through the interaction of βC1 with AGO1 (Eini, 2017). Additionally, several βC1s have been demonstrated to suppress host‐mediated transcriptional and PTGS processes.

### Suppression of post‐transcriptional gene silencing

The PTGS machinery, which involves sequence‐specific degradation of double‐stranded foreign RNA, serves as a robust and conserved mechanism employed by the plants to fight against pathogenic viruses, transgenes and transposons (Pumplin and Voinnet, 2013). In the process of plant‐virus interaction, double‐stranded replicative intermediates and overlapping segments of mRNA transcripts of RNA and DNA viruses, respectively, become the targets of the RNA silencing machinery. These targets are processed by DICER‐like proteins (DCL) to dsRNAs of 21–24 nucleotides (Voinnet, 2001), known as siRNAs. These siRNAs are recruited by ARGONAUTE (AGO) proteins to form a nuclease cleaving complex called RNA‐induced silencing complex (RISC) that guides binding and cleaving of homologous transcripts of viral pathogens (Hammond *et al*., 2000). Either viral RNAs or their cleavage products serve as templates for host RNA‐dependent RNA‐polymerases (RDRs) and yield dsRNAs that after being cleaved by DCLs produce secondary siRNAs.

To counter PTGS of the host, plant viruses encode proteins that suppress the gene silencing at various junctures of the silencing pathway (Pumplin and Voinnet, 2013; Roth *et al*., 2004). The viral pathogenicity determinant proteins usually act as the silencing suppressors. In addition to helper virus‐encoded AC2/C2, AC4/C4, and AV2/V2 proteins, the βC1 proteins, encoded by betasatellites associated with different begomoviruses like TYLCCNV, *Tomato leaf curl Java virus, Bhendi yellow vein mosaic virus *(BYVMV)*, Cotton leaf curl Multan virus* (CLCuMuV) etc. function as the extra silencing suppressor molecules (Cui *et al*., 2005; Gopal *et al*., 2007; Kon *et al*., 2007). Thus, βC1 proteins enhance the helper virus accumulation and the severity of infection. However, different βC1 proteins share limited sequence similarity amongst them, a fact that suggests the plurality in the mechanism of how different βC1 proteins interfere with the silencing pathway of the host (Briddon *et al*., 2001). Nuclear localization signal (NLS) of TYLCCNB‐βC1 is essential for its nuclear localization and suppression of gene silencing activity (Cui *et al*., 2005). When expressed transgenically, TYLCCNB‐βC1 overexpressed plants developed virus‐like symptoms indicating the protein’s ability to cause developmental abnormalities in the plant. The developmental process of a plant is influenced by the microRNAs (miRNAs) that regulate the host gene expressions. Ranging in size from 20 to 24 nucleotides, the miRNAs are non‐coding single‐stranded small RNAs that target specific mRNAs for cleavage or translational inhibition and thus act as the master modulators of gene expression at the mRNA level (Bartel 2009). As both siRNA mediated gene silencing and miRNA biogenesis pathways require the nuclear localized DICER‐like proteins, the point of interference of βC1 with the silencing pathway is likely present at the initial stage of maturation of miRNA (Xie *et al*., 2004). However, *Cotton leaf curl* *Multan betasatellite* encoded βC1 (CLCuMuB‐βC1) possesses suppressor activity despite lacking an NLS (Amin *et al*., 2011; Tiwari *et al*., 2013).

Studies have revealed that a plant itself encodes the endogenous suppressors of RNA silencing (ESRs) for proper regulation of the gene silencing machinery (Anandalakshmi *et al*., 2000). βC1, as the pathogenicity factor, modulates the host ESRs to thwart host defence mediated by RNA silencing. TYLCCNB‐βC1 acts as a viral suppressor by up‐regulating the expression of *N.* *benthamiana* calmodulin‐like protein, Nbrgs‐CaM (Fig. 2), which in turn, represses the level of both RDR6 and secondary siRNAs causing suppression of gene silencing (Li *et al*., 2014b). Moreover, since βC1 expression suppresses viral siRNA production by an RDR6 independent pathway, it becomes clear that βC1 imparts a pleiotropic effect on the host RNA silencing machinery (Li *et al*., 2014a).

Autophagy is reported to be an important antiviral defence mechanism. In plants, suppression of autophagy‐related genes (*ATGs)* expression results in diminished vitality and disease resistance. Interestingly, reports suggest that viruses employ strategies to use autophagy for their own propagation with autophagy proteins acting as the proviral factors (Dreux and Chisari 2009).  In a recent study, the interaction between calmodulin‐like protein (NbCaM) and suppressor of gene silencing 3 (SGS3) proteins has been shown to lead to phosphatidylinositol 3‐kinase complex mediated degradation of SGS3. The class III phosphatidylinositol 3‐kinase is involved in the initiation of autophagy, and degradation of SGS3 subsequently facilitates the infection by the geminiviruses TYLCCNV and TYLCCNB (Li *et al*., 2017a). As NbCaM promotes geminivirus infection via the autophagy pathway and βC1 induces up‐regulation of NbCaM, the proviral function of autophagy in geminivirus infection might also be dependent on the presence of betasatellite (Li *et al*., 2017a).

### Suppression of transcriptional gene silencing

In plants, TGS is an epigenetic phenomenon that involves repressive histone modifications and RNA‐directed DNA methylation (RdDM). RdDM not only serves as a regulator of endogenous gene expression but also acts as an effective tool to repress the genes of the DNA viruses (Matzke *et al*., 2009). Geminiviruses do not encode polymerase and they depend on the host machinery for replication and transcription. Within the nucleus of an infected plant cell, geminivirus single‐stranded genomic DNA is converted to double‐stranded DNA, and associates with histone to form minichromosomes, which function as the template for replication and transcription (Kushwaha *et al*., 2017; Paprotka *et al*., 2015; Pilartz and Jeske, 2003). These minichromosomes become the target of the TGS machinery of the plant. TGS and PTGS processes complement each other to raise the antiviral defence by specifically inactivating viral RNAs resulting in reduced virus replication, hypermethylation of viral genomes and subsequent symptom disappearance (Raja *et al*., 2010). The pattern in which a viral genome becomes the subject to methylation is specific for a particular virus‐host combination (Chellappan *et al*., 2004). As the plants target the regulatory elements of viral genome for TGS mechanism, the intergenic regions, in addition to the coding regions of Rep, REn, TrAP and MP genes, seems to be the ‘hot spots’ for methylation (Rodriguez‐Negrete *et al*., 2009; Yang *et al*., 2011b).

As a counter‐defence mechanism, plant DNA viruses code for proteins that can serve as suppressors of TGS working at different stages of the process (Hohn and Vazquez, 2011). The protein βC1 has been demonstrated to suppress the methylation‐mediated RNA silencing. TYLCCNB‐βC1 acts as a suppressor of TGS by targeting the enzyme S‐adenosyl homocysteine hydrolase (SAHH) and inhibiting the synthesis of S‐adenosyl‐methionine (Fig. 2), the methyl group donor for DNA methylation (Yang *et al*., 2011b). Further, in *N. benthamiana*, TYLCCNB changes the methylation pattern of both the helper virus promoter and the intergenic region, as well as the host genome. The level of methylation at both CG and non‐CG sites were substantially reduced by βC1 expression (Yang *et al*., 2011b). In this case, too, disruption of the NLS of βC1 hampered its ability to interact with SAHH and the TGS reversal was suppressed. However, plant immunity has evolved to deploy host SUCROSE‐NONFERMENTING1‐related kinase (SnRK1) protein to overcome βC1 mediated suppression of TGS. SnRK1 mediated phosphorylation of βC1 protein reduces its TGS suppressor function without affecting its stability, self‐interaction, subcellular localization and interaction with ASYMMETRIC LEAVES1 (AS1) transcription factor (Zhong *et al*., 2017).

### Implication of the interaction of βC1 and host ubiquitin‐proteasome machinery on disease development

The central role of the ubiquitin‐proteasome system is to degrade the redundant/misfolded cellular proteins and the regulatory proteins with short half‐lives (cell cycle regulators, transcription factors, signal transducer, etc.). Ubiquitination of protein substrate is mediated by the serial action of E1 (ubiquitin‐activating enzyme), E2 (ubiquitin‐conjugating enzyme), and E3 (ubiquitin‐ligase) proteins (Stone *et al*., 2005). The proteins that are polyubiquitinated at their Lys‐residues are recognized and degraded by the host ubiquitin‐proteasome system. Plants defence mechanisms had evolved to deploy host proteasomal degradation machinery to degrade the viral and cellular proteins that contribute to the regulation of viral infections (Verchot, 2016). Targeting and exploiting the host ubiquitin system to invade the host cell machinery is a strategy used by different plant viruses (Alcaide‐Loridan and Jupin, 2012). In recent studies, βC1 protein has been found to aid in the stabilization of viral proteins and the establishment of infection by modulating a component of the plant ubiquitin‐proteasome system. A yeast two‐hybrid screening revealed that *Nicotiana tabacum* RING‐finger protein NtRFP1 interacts with TYLCCNV‐βC1. NtRFP1, being a functional E3 ubiquitin‐ligase, polyubiquitinates and degrades the βC1 protein by 26S proteasome‐mediated degradation (Shen *et al*., 2016). During betasatellite infection, the up‐regulation of NtRFP1 and subsequent degradation of the pathogenicity factor by the ubiquitin/26S proteasome system results in developing resistance against geminivirus infection.

The tomato SUCROSE‐NONFERMENTING1‐related kinase (SlSnRK1) interacts with and phosphorylates the TYLCCNB‐βC1 protein and this probably leads to its proteasomal degradation (Shen *et al*., 2011). The screening of a tomato yeast two‐hybrid library with CLCuMuB‐βC1 identified a host interacting factor namely *Solanum lycopersicum* ubiquitin‐conjugating enzyme 3 (SlUBC3) (Fig. 2), a novel ubiquitin‐conjugating enzyme (Eini *et al*., 2009b). The interaction of βC1 with SlUBC3‐E2 protein correlates with the compromised polyubiquitination of host proteins in the betasatellite infected plants. Further, interaction studies confirmed that βC1 protein of ToLCV‐associated betasatellites also can interact with the SlUBC3‐E2 protein (Eini *et al*., 2009b). Additionally, CLCuMuB‐βC1 interacts with S‐phase kinase‐associated protein (SKP1) and interferes with SKP1‐CUL1 interaction; this subsequently prevents the formation of plant SCF‐E3 ubiquitin‐ligase complex (SKP1/CUL1/FBX/RBX1) (Jia *et al*., 2016). The βC1 protein‐mediated inhibition of the ubiquitin‐conjugating system enhances the CLCuMV infection and accumulation of viral DNA in the infected plant. Therefore, the interaction of βC1 with components of the plant’s ubiquitin‐proteasome system appears to be a crucial aspect of the betasatellite pathogenicity.

## β**C1 Mitigates the Antiviral Resistance Established by Chloroplast and Plant Defence** Responsive** Hormones**

**Figure 3:**
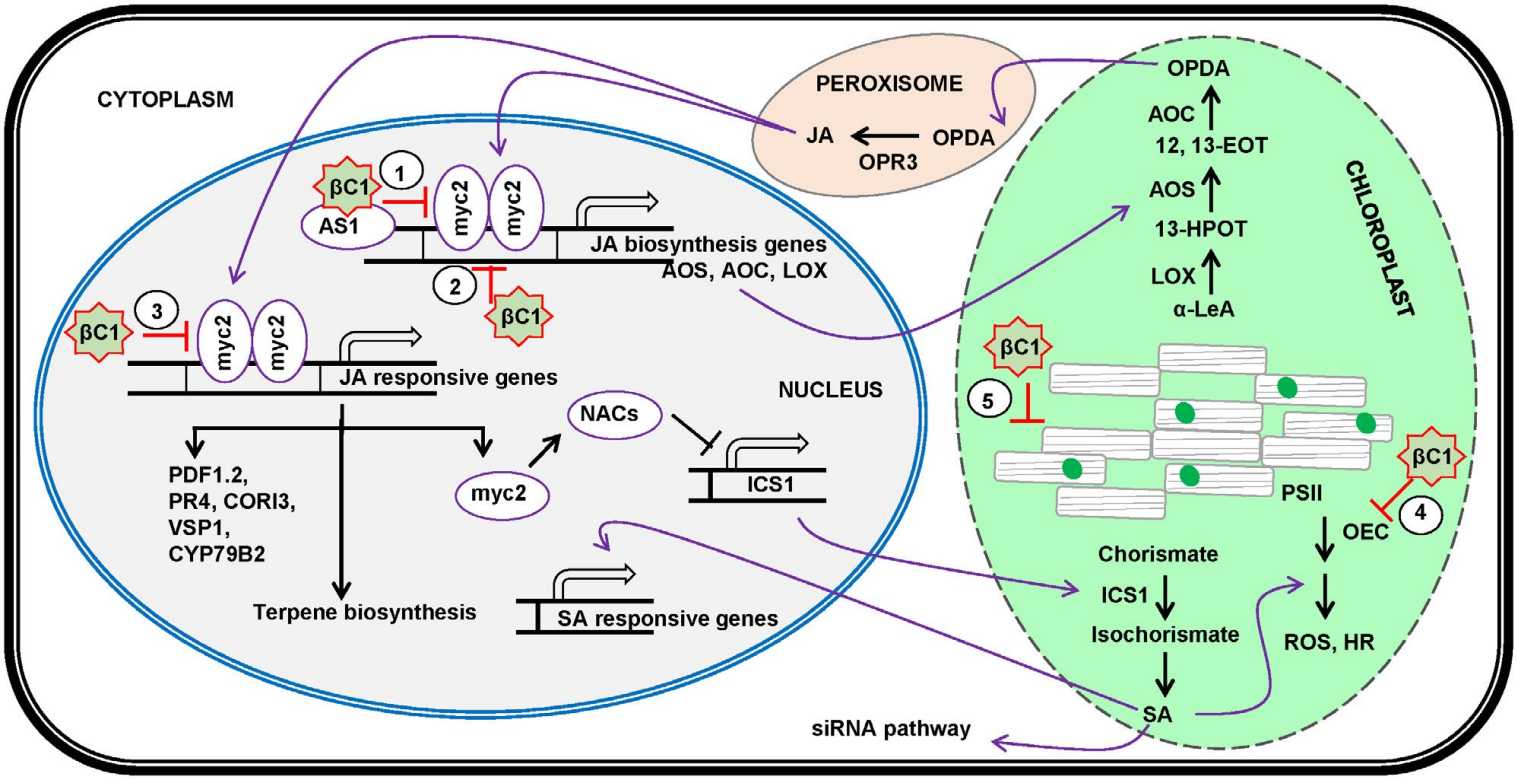
Hypothetical working model illustrating βC1‐mediated counter‐defence against plant defence responsive hormones. Nuclear‐encoded chloroplast localized allene oxide synthase (AOS), allene oxide cyclase (AOC) and lipoxygenase (LOX) are key enzymes of JA synthesis pathway. The chloroplast membrane synthesized the polyunsaturated fatty acid, α‐linolenic acid (18:3) (α‐LeA), which is converted to 13(S)‐hydroperoxy‐octadecatrienoic acid (13‐HPOT) by the reaction catalyzed by LOX. AOS catalyzes the conversion of 13‐HPOT to 13(S)‐epoxy‐octadecatrienoic acid (12,13‐EOT) and further to 12‐oxophytodienoic acid (OPDA) by AOC. OPDA, being involved in the production of jasmonic acid (JA), is transported to peroxisome and converted to JA by the action of OPDA reductase 3 (OPR3). JA activates JAZ proteins, and other JA‐responsive genes, such as MYC2, PDF1.2, PR4, CORI3, VSPI, CYP79B2, etc. The MYC2 transcription factor activates the JA biosynthetic genes, terpene biosynthesis genes, and suppress the salicylic acid (SA) synthesis by down‐regulating the expression of isochorismate synthase 1 (ICS1) through activating NAC transcription factor. In an infected plant cell, βC1 protein modulates the JA‐mediated defence response by targeting the MYC2 transcription factor. Interaction of βC1 with ASYMMETRIC LEAVES 1 (AS1) transcription factor suppresses the expression of MYC2, which is required for the synthesis of JA biosynthetic gene (1). βC1 protein also interacts and interferes with MYC2 promoter binding activity leading to the suppression of JA‐biosynthesis genes expression (2) and JA‐responsive gene (3). In chloroplast, nuclear‐encoded ICS catalyzes the conversion of chorismate to isochorismate and facilitate the production of SA. SA mediates the defence against biotrophic pathogens by activating SA‐responsive genes, siRNA pathway, and defence‐related reactive oxygen species production (ROS). Geminivirus‐βC1 protein localizes into chloroplast and interferes with PSII electron transport rate by affecting its oxygen‐evolving complex. βC1 mediated interference of PSII electron transport rate lead to intervenes in ROS production (4). The localization of βC1 in the chloroplast and disruption of its ultrastructure facilitate the optimal niche for virus probably by affecting the nucleus‐chloroplast signalling and the plant defence hormone (SA, JA) production (5). HR refers to hypersensitive response.

Being obligate parasites, plant viruses exploit the host cellular machinery for their genome replication, protein synthesis, intracellular and systemic movement. The plant organelle chloroplast, itself being a chimera of bacterial, viral and plant components (Zhao *et al*., 2016), is a potential target for plant‐virus interaction. Besides its role in photosynthesis, chloroplasts have crucial functions in the defence mechanisms of the plants against viruses and other biotrophic and necrotrophic pathogens (Haxim *et al*., 2017). Chloroplasts contribute to the defence response by providing the site for the production of SA, JA and ROS (Ascencio‐Ibanez *et al*., 2008; Bowling *et al*., 1994; Nomura *et al*., 2012). Further, the antagonistic regulation of SA and JA biogenesis and signalling is accomplished by the components of the chloroplast (Fig. 3). The calcium spike signal, triggered during non‐compatible plant‐pathogen interactions, is perceived by *CALCIUM‐SENSING* *RECEPTOR* (CAS) protein localized on the thylakoid membrane of the chloroplast. The SA accumulation, driven by the activation of CAS protein receptor illustrates the association of chloroplast with nuclear and cytoplasmic immune responses (Nomura *et al*., 2012).

Geminivirus infection causes up‐regulation of several genes associated with SA biosynthesis and signalling (Ascencio‐Ibanez *et al*., 2008). Elevated transcript level of SA‐responsive marker genes was found in the plants infected with Cabbage leaf curl virus (CaLCuV). In addition, the expression of transcription factors downstream of SA response pathways such as TGA1, TGA3, TGA5 and WRKY70 was up‐regulated during geminivirus infection (Ascencio‐Ibanez *et al*., 2008). *CONSTITUTIVE EXPRESSOR* *OF PR GENES* (CPR1), an F‐box protein, has been shown to negatively regulate SA production (Gou *et al*., 2009). The *crp1*‐mutant plant showed constitutively elevation of SA and its responsive gene and had elevated resistance to CaLCuV infection (Ascencio‐Ibanez *et al*., 2008). The jasmonate signalling, as a part of the defence response of the plant, interrupts the geminivirus propagation and proliferation. *Arabidopsis thaliana* plants treated with exogenous jasmonate showed reduced susceptibility and DNA accumulation while challenged with *Beet curly top virus* (Lozano‐Duran *et al*., 2011).

*MITOGEN‐ACTIVATED PROTEIN KINASE 3* (MAPK3) also contributes to tolerance against *Tomato yellow leaf curl virus* infection through SA/JA signalling mediated defence response (Li *et al*., 2017b).

Extensive studies on the biological function of βC1 protein clarified its role in suppressing the plant defence response by disrupting the chloroplasts (Bhattacharyya *et al*., 2015). Interestingly, TYLCCNB‐βC1 protein was detected in the nucleus as well as in the chloroplast of the infected *N. benthamiana* cells by immunoelectron microscopy (Cui *et al*., 2005). *Radish leaf curl betasatellite* (RaLCB) infection or mere transient expression of βC1 protein perturbed the chlorophyll pigment content, reduced the photosynthetic efficiency, resulting in inappropriate accumulation of the photoassimilates, and altered the expression of nuclear‐encoded chloroplastic proteins. The RaLCB‐βC1 protein was demonstrated to localize into the chloroplasts of the infected plant, affecting the ultrastructure and photosynthetic function of the organelle (Bhattacharyya *et al*., 2015). Inhibition of host photosynthesis might provide an optimal microenvironment for plant viruses (Bhattacharyya and Chakraborty, 2018). Also, considering the importance of chloroplasts in the defence response of the plants, the βC1‐mediated disruption of chloroplast structure during betasatellite infection might be a part of the viral counter‐defence strategy that hampers the elevation of plant defence hormones. TYLCCNB‐βC1 protein functions as a pathogenicity factor by selective suppression of JA‐responsive genes (Fig. 3). The expression of JA‐biosynthesis genes (FAD3 and FAD7) and JA‐responsive genes (PR4, PDF1.2, VSP1, CORI3 and CYP79B2) were repressed in the betasatellite infected plants (Fig. 3) (Li *et al*., 2014b; Zhang *et al*., 2012). The suppression of JA response by βC1 is accomplished by its interaction with AS1 protein in the molecular disguise of *ASYMMETRIC LEAVES2* (AS2) protein (Yang *et al*., 2008). MYC2, a basic helix‐loop‐helix transcription factor is a key downstream component of JA signalling (Li *et al*., 2014b). TYLCCNB‐βC1 interacts and interferes with the dimerization of MYC2, which is necessary for binding to G‐box/G‐box‐like motif present in its promoter (Stone *et al*., 2005). Various studies showed that JA generates plant defence responses against geminivirus infection (Sun *et al*., 2017). JA exhibits its responses by binding to its receptor, SCF^col1 ^complex. Recently, CLCuMuB‐βC1 has been shown to hinder the JA signalling pathway in the plant through the interaction with SKP1 protein that impairs SCF^col1^. As JA is likely involved in anti‐virus defence, suppression of JA signalling resulted in enhanced viral accumulation and symptoms in plants (Jia *et al*., 2016).

## βC1 Protein Aids Movement of Helper Begomoviruses in their Hosts

The DNA‐B encoded NSP and MP mediate local and systemic movements of the bipartite viruses, respectively, inside the hosts (Gafni and Epel, 2002). According to the existing models describing the mechanism of systemic movement of the bipartite viral DNA in the host, functional complementarity between MP and NSP is a necessary condition. As proposed by Noueiry *et al*. (1994), NSP aids the intracellular movement of the viral genome from the nucleus to the cytoplasm, and MP transports the viral cargo from cytoplasm through plasmodesmata helping the cell‐to‐cell movement of the virus. In an alternative model of movement, MP mediates the NSP‐DNA complex in both intra‐ and intercellular movement (Lazarowitz and Beachy, 1999). The ability of host histone H3 to specifically interact with NSP and MP implicates its role in the transport of geminiviral DNA complex from the nucleus to the cytoplasm as well as in cell‐to‐cell transport through plasmodesmata (Zhou *et al*., 2011). However, a monopartite geminivirus, in absence of DNA‐B encoded MP and NSP, essentially needs an alternative strategy for the systemic and cell‐to‐cell movement. Interestingly, a number of betasatellites associated with diverse monopartite begomoviruses have been suggested to complement the movement function of DNA‐B encoded proteins of different geminiviruses (Saeed *et al*., 2007; Saunders *et al*., 2000). Although ToLCNDV DNA‐A alone induces local but not systemic infection, the presence of CLCuMuB facilitates the systemic infection of ToLCNDV DNA‐A in tomato. CLCuMuB with disrupted βC1 failed to help ToLCNDV DNA‐A in such systemic movements (Patil and Fauquet, 2010; Saeed *et al*., 2007).

Many of the βC1 proteins of different betasatellites have either an NLS or nuclear export signal (NES) (Kumar *et al*., 2006). The possibility of a correlation between symptom induction and intracellular movement of βC1 became stronger as the nuclear localization of TYLCCNB‐βC1 was found to be crucial for symptom development in *N. benthamiana* (Cui *et al*., 2005). Further studies revealed that βC1 protein interacts with the CP of helper virus and also with the host nuclear importin like protein karyopherin α. βC1 encoded by *Bhendi yellow vein mosaic betasatellite* (BYVMB) possesses a strong NES and also physically interacts with BYVMV encoded CP, which lacks NES (Kumar *et al*., 2006). Such complementarity enables the interacting partners in nuclear export and import, and is suggested to be a process analogous to the interaction model of NSP and MP related to the nuclear transport and cell‐to‐cell movement of bipartite viruses (Gafni and Epel, 2002). Nevertheless, the βC1 deletion‐mutant of TYLCCNB‐Y10β was capable of moving systemically in the plant and underwent encapsidation by the helper virus (Qian and Zhou, 2005).Thus, βC1 is considered to have a dispensable role in the systemic movement of a monopartite virus.

## Role of Betasatellite in the Tripartite Interaction of Host‐Vector‐Virus

The complex tripartite interaction amongst host plant, insect vector and infecting virus produce the final outcome of the infection (Sun *et al*., 2017). Recent studies demonstrated that B biotype of *Bemisia tabaci*, the whitefly responsible for begomovirus transmission participates in a synergistic relationship with the virus resulting in increased fecundity of the insect in TYLCCNV infected plants (Luan *et al*., 2013). Begomoviruses alter the host’s nutritional profile and defence responses to make the host more attractive to the vector (Luan *et al*., 2014). Wounding and herbivore attack on plants induce JA signalling, a major defence pathway against insects (Galis *et al*., 2009). Betasatellites manipulate the anti‐herbivore response of the plant by interfering with JA signalling pathway through various strategies (Li *et al*., 2014b; Yang *et al*., 2008).

Firstly, βC1 down‐regulates expression of JA‐responsive genes by intervening with the AS1/AS2 complex formation (Fig. 2) (Yang *et al*., 2008). The other approach that betasatellite adopts in this context is to suppress the synthesis of organic volatile monoterpenes, α‐bergamotene and β‐myrcene in the plant. Some of the volatile compounds derived from plants, like linalool, eugenol, myrcene, limonene and 1, 8‐cineole, are involved in evoking positive behavioural responses from female *B. tabaci* of B biotype (Feng‐Qin, 2008). βC1 protein of TYLCCNB interacts directly with MYC2 transcription factor of *A. thaliana* and *N. benthamiana* (Li *et al*., 2014b). The physical interaction of βC1 with MYC2 leads to inhibition of the dimerization and the DNA binding capability of the MYC2, which affects the induction of terpene synthase gene. Reduced synthesis and emission of α‐bergamotene and β‐mycrene attract more vectors to infect and lay eggs on the virus infected plants (Li *et al*., 2014b). Interference of βC1 with the function of a master regulator transcription factor‐like MYC2 is highly significant for the physiology and cellular responses of the plant. Furthermore, genes related to indole and aliphatic glucosinolates, two components involved in the herbivore‐induced response, were down‐regulated in βC1‐overexpressed plants (Li *et al*., 2014b).

## Conclusions and Future Perspectives

The threats of geminivirus infection on economically important crops aggravates due to the association of betasatellites with the majority of monopartite begomoviruses. Plants generate defence responses against geminivirus infection by activating diverse mechanisms such as RNA silencing, ubiquitin/proteasome‐related protein degradation system, autophagy, chloroplast machinery and innate immunity mediated by several host factors. The βC1 protein plays a multitasking role as it suppresses the gene silencing, attenuates plant defence responses, induces disease symptoms and possibly helps in the virus movement. The βC1 protein being a strong silencing suppressor facilitates the viral pathogenesis by interfering with host PTGS and TGS machinery. βC1 aggravates the symptoms by stabilizing the viral proteins. βC1 protein interacts with ubiquitin‐conjugating enzyme, E2 and thereby exploits the ubiquitin‐proteasome system of the plant leading to compromised polyubiquitination of proteins.

Chloroplast accomplishes the defence response against viruses by facilitating the autophagy‐mediated viral protein degradation, production of defence‐related ROS and contributing to the production of SA and JA. As a viral counter‐defence strategy against chloroplast‐mediated immunity response, βC1 localizes into chloroplasts and disrupts the structure and function of the chloroplasts. Additionally, betasatellite interferes with plant anti‐herbivore response, attracts the insect vector for transmission and thereby supports the viral transmission into a new host. The βC1 protein adopts two independent strategies to interfere with JA response, either by βC1‐AS1 interaction or by βC1‐MYC2 interaction. In the future, investigating plant‐geminivirus betasatellite interactions, in terms of the performance of photosystem, the fate of chloroplast, and the autophagy would help in deepening the understanding of the molecular mechanisms of βC1 mediated pathogenesis. Further, understanding of βC1 mediated regulation of the defence hormones of the plant would help to develop better strategies against geminiviral diseases.

## Supporting information


**Table S1** Geographical distribution of helper virus betasatellite disease complexes across plant species.
