# Supplementary material for: Multifaceted role of geminivirus associated betasatellite in pathogenesis
Source: Mol Plant Pathol. 2019 Jun 18;20(7):1019–33. doi: 10.1111/mpp.12800 (PMC6589721; doi:10.1111/mpp.12800)
Supplement: Supplementary file 1 — Table S1 Geographical distribution of helper virus betasatellite disease complexes across plant species. [file MPP-20-1019-s001.docx]

**Table S1. Geographical distribution of helper virus-betasatellite disease complexes across plant species`**

| **S.N0** | **Betasatellite** | **Abbreviated name** | **Accession Number** | **Associated helper begomovirus** | **Geographical location** | **Infected host** | **Family** |
| --- | --- | --- | --- | --- | --- | --- | --- |
| 1 | *French bean leaf curl betasatellite* | FBLCuB | JQ866298 | *French bean leaf curl virus* | India | *Phaseolus vulgaris* | Fabaceae |
| 2 | *Papaya leaf curl India betasatellite* | PaLCuINB | HM143906 | Not identified | India | *Carica papaya* | Caricaceae |
| 3 | *Mungbean yellow mosaic betasatellite* | MYMB | JX443646 | *Mungbean yellow mosaic India virus* | India | *Vigna unguiculata* | Fabaceae |
| 4 | *Rhynchosia yellow mosaic betasatellite* | RhYMB | KP752092 | Not identified | India | *Phaseolus vulgaris* | Fabaceae |
| 5 | *Papaya leaf curl China betasatellite* | PaLCuCNB | KJ642219 | Not identified | China | *Carica papaya* | Caricaceae |
| 6 | *Tomato leaf curl China betasatellite* | ToLCCNB | AJ704609 | Not identified | China | *Solanum lycopersicum* | Solanaceae |
| 7 | *Pea leaf distortion betasatellite* | PLDiB | KY001644 | *Pea leaf distortion virus* | Nepal | *Pisum sativum* | Fabaceae |
| 8 | *Tomato leaf curl Laos betasatellite* | ToLCLAB | AJ542491 | Not identified | Laos | *Solanum*  *lycopersicum* | Solanaceae |
| 9 | *Malvastrum leaf curl Guangdong betasatellite* | MaLCuGuB | KF912951 | Not identified | China | *Malvastrum coromandelianum* | Malvaceae |
| 10 | *Tomato leaf curl Laguna betasatellite* | ToLCLaB | AB307732 | *Tomato leaf curl Philippines virus-Los Banos* | Philippines | *Solanum*  *lycopersicum* | Solanaceae |
| 11 | *Tomato leaf curl Philippine betasatellite* | ToLCPHB | AB308071 | *Tomato leaf curl Philippines virus* | Philippines | *Solanum*  *lycopersicum* | Solanaceae |
| 12 | *Malvastrum leaf curl betasatellite* | MaLCuB | AM072289 | *Malvastrum leaf curl virus* | China | *Malvastrum coromandelianum* | Malvaceae |
| 13 | *Hedyotis yellow mosaic betasatellite* | HYMB | KF641186 | *Hedyotis uncinella yellow mosaic virus* | Vietnam | *Hedyotis uncinella* | Rubiaceae |
| 14 | *Tomato leaf curl Java virus betasatellite* | ToLCJaB | KC282642 | Not identified | Nepal | *Carica papaya* | Caricaceae |
| 15 | *Ageratum yellow vein betasatellite* | AYVB | AJ252072 | *Ageratum yellow vein virus* | Singapore | *Ageratum conyzoide* | Asteraceae |
| 16 | *Alternanthera yellow vein betasatellite* | AlYVB | DQ641716 | *Alternanthera yellow vein virus* | Vietnam | *Zinnia elegans* | Asteraceae |
| 17 | *Tobacco curly shoot betasatellite* | TobCSB | AJ421484 | Not identified | China | *Nicotiana tabacum* | Solanaceae |
| 18 | *Tomato leaf curl Nepal betasatellite* | ToLCNPB | AJ542492 | Not identified | Nepal | *Solanum*  *lycopersicum* | Solanaceae |
| 19 | *Tomato yellow leaf curl Thailand betasatellite* | TYLCTHB | AJ566746 | *Tomato leaf curl Philippines virus* | China | *Solanum*  *lycopersicum* | Solanaceae |
| 20 | *Rose leaf curl betasatellite* | RoLCuB | GQ478344 | *Rose leaf curl virus* | Pakistan | *Rosa chinensis* | Rosaceae |
| 21 | *Tobacco leaf curl betasatellite* | TobLCuB | AM260465 | Not identified | Pakistan | *Pedilanthus tithymaloides* | Euphorbiaceae |
| 22 | *Mirabilis leaf curl betasatellite* | MiLCuB | LK054803 | Not identified | India | *Mirabilis jalapa* | Nyctaginaceae |
| 23 | *Tomato leaf curl Patna betasatellite* | ToLCPaB | EU862324 | *Tomato leaf curl Patna virus* | India | *Solanum*  *lycopersicum* | Solanaceae |
| 24 | *Chili leaf curl Jaunpur betasatellite* | ChLCuJB | HM007103 | *Chili leaf curl virus* | India | *Capsicum annuum* | Solanaceae |
| 25 | *Tomato leaf curl Joydebpur betasatellite* | ToLCJoB | AJ966244 | Not identified | Bangladesh | *Solanum*  *lycopersicum* | Solanaceae |
| 26 | *Tomato leaf curl Gandhinagar betasatellite* | ToLCGanB | KC952006 | *Tomato leaf curl Gandhinagar virus* | India | *Solanum lycopersicum* | Solanaceae |
| 27 | *Tomato leaf curl betasatellite* | ToLCB | AJ316036 | Not identified | Pakistan | *Solanum lycopersicum* | Solanaceae |
| 28 | *Chili leaf curl betasatellite* | ChLCuB | AJ316032 | *Pepper leaf curl Lahore virus* | Pakistan | *Capsicum annuum* | Solanaceae |
| 29 | *Tomato yellow leaf curl Rajasthan betasatellite* | ToLCRaB | AY438558 | Not identified | India | Not available | Not available |
| 30 | *Ageratum yellow leaf curl betasatellite* | AYLCB | AJ316026 | *Ageratum yellow vein virus* | Pakistan | *Ageratum conyzoide* | Asteraceae |
| 31 | *Chili leaf curl Sri Lanka betasatellite* | ChLCuSLB | JN638445 | *Chili leaf curl Sri Lanka virus* | Sri Lanka | *Capsicum annuum* | Solanaceae |
| 32 | *Tomato leaf curl Bangalore betasatellite* | ToLCBaB | AY428768 | *Tomato leaf curl Bangalore virus* | India | *Solanum lycopersicum* | Solanaceae |
| 33 | *Papaya leaf curl betasatellite* | PaLCuB | AY244706 | *Papaya leaf curl virus* | India | *Carica papaya* | Caricaceae |
| 34 | *Croton yellow vein mosaic betasatellite* | CroYVMB | AM410551 | *Croton yellow vein virus* | Pakistan | *Croton bonplandianus* | Euphorbiaceae |
| 35 | *Tomato leaf curl Sri Lanka betasatellite* | ToLCSLB | AJ542493 | Not identified | Sri Lanka | *Solanum*  *lycopersicum* | Solanaceae |
| 36 | *Ageratum yellow vein India betasatellite* | AYVINB | AJ557441 | Not identified | India | *Ageratum conyzoide* | Asteraceae |
| 37 | *Ageratum yellow vein Sri Lanka betasatellite* | AYVSLB | AJ542498 | *Ageratum yellow vein virus* | Sri Lanka | *Ageratum conyzoide* | Asteraceae |
| 38 | *Andrographis yellow vein leaf curl betasatellite* | AnYVLCuB | KC967282 | *Eclipta yellow vein virus* | India | *Andrographis paniculata* | Acanthaceae |
| 39 | *Tomato yellow leaf curl Yunnan betasatellite* | ToYLCYnB | KF640694 | *Tomato yellow leaf curl China virus* | China | *Nicotiana tabacum* | Solanaceae |
| 40 | *Tomato yellow leaf curl China betasatellite* | TYLCCNB | AJ420313 | *Tomato yellow leaf curl China virus* | China | *Nicotiana tabacum* | Solanaceae |
| 41 | *Tomato yellow leaf curl Shandong betasatellite* | ToYLCShB | KP322555 | Not identified | China | *Solanum*  *lycopersicum* | Solanaceae |
| 42 | *Tomato yellow leaf curl Vietnam betasatellite* | TYLCVNB | DQ641714 | *Tomato yellow leaf curl Vietnam virus* | Vietnam | *Solanum*  *lycopersicum* | Solanaceae |
| 43 | *Tomato yellow leaf curl Yunnan betasatellite isolate* YN4296 | ToYLCYnB-YN4296 | KX881360 | Not identified | China | *Amaranthus blitum* | Amaranthaceae |
| 44 | *Tomato leaf curl Malaysia betasatellite* | ToLCMYB | KM051528 | Not identified | Malaysia | *Solanum*  *lycopersicum* | Solanaceae |
| 45 | *Sida leaf curl virus-associated DNA beta* | SiLCuB | AM050732 | *Sida leaf curl virus* | China | *Sida cordifolia* | Malvaceae |
| 46 | *Leucas zeylanica yellow vein virus satellite DNA beta* | LYVB | GQ421324 | Not identified | Sri Lanka | *Leucas zeylanica* | Lamiaceae |
| 47 | *Ageratum leaf curl Buea betasatellite* | ALCuBB | FR717140 | *Ageratum leaf curl Cameroon virus* | Cameroon | *Ageratum conyzoide* | Asteraceae |
| 48 | *Ageratum leaf curl Cameroon betasatellite* | ALCuCMB | FM164737 | Ageratum leaf curl Cameroon virus | Cameroon | *Ageratum conyzoide* | Asteraceae |
| 49 | *Tomato leaf curl Togo betasatellite*-[Togo:2006] isolate GH-Ago3-12 | ToLCTGB | KT382328 | Not identified | Ghana | *Solanum lycopersicum* | Solanaceae |
| 50 | *Momordica yellow mosaic betasatellite* | MamYMB | KT454829 | Not identified | Benin | *Momordica charantia* | Cucurbitaceae |
| 51 | *Okra leaf curl Oman betasatellite* | OLCuOMB | KF267444 | *Okra leaf curl Oman virus* | Oman | *Abelmoschus esculentus* | Malvaceae |
| 52 | *Cotton leaf curl Gezira betasatellite* | CLCuGeB | DQ644564 | Not identified | Sudan | *Datura stramonium* | Solanaceae |
| 53 | *Tomato leaf curl Yemen betasatellite* | ToLCYEB | JF919717 | *Tomato leaf curl Sudan virus* | Yemen | *Nicotiana tabacum* | Solanaceae |
| 54 | *Cotton leaf curl Multan betasatellite* | CLCuMuB | AJ298903 | *Cotton leaf curl virus* | Pakistan | *Gossypium hirsutum* | Malvaceae |
| 55 | *Bitter gourd leaf curl disease-associated DNA beta* | BiLCuB | AY817151 | Not identified | India | *Momordica charantia* | Cucurbitaceae |
| 56 | *Bhendi yellow vein mosaic betasatellite* | BYVB | AJ308425 | *Bhendi yellow vein mosaic virus* | India | *Abelmoschus esculentus* | Malvaceae |
| 57 | *Cardiospermum yellow leaf curl betasatellite* | CaYLCuB | AM933578 | *Cardiospermum yellow leaf curl virus* | Sri Lanka | *Cardiospermum microcarpum* | Sapindaceae |
| 58 | *Eupatorium yellow vein betasatellite* | EpYVB | AJ438938 | *Eupatorium yellow-vein virus* | Japan | *Eupatorium makinoi* | Asteraceae |
| 59 | *Eupatorium yellow vein mosaic betasatellite* | EpYVV | AB300464 | *Eupatorium yellow-vein virus* | Japan | *Eupatorium makinoi* | Asteraceae |
| 60 | *Honeysuckle yellow vein betasatellite* | HYVB | AJ316040 | *Honeysuckle yellow vein mosaic virus* | United Kingdom | *Lonicera japonica* | Caprifoliaceae |
| 61 | *Honeysuckle yellow vein mosaic betasatellite* | HYVMB | AB182263 | Not identified | Japan | *Lonicera japonica* | Caprifoliaceae |
| 62 | *Tobacco leaf curl Japan betasatellite* | TbLCJRB | AB236324 | *Honeysuckle yellow vein mosaic virus* | Japan | *Solanum lycopersicum* | Solanaceae |
| 63 | *Siegesbeckia yellow vein betasatellite* | SiYVB | KF499590 | *Siegesbeckia yellow vein virus* | China | *Sigesbeckia glabrescens* | Asteraceae |
| 64 | *Vernonia yellow vein betasatellite* | VYVB | FN435836 | *Vernonia yellow vein virus* | India | *Vernonia cinerea* | Asteraceae |
| 65 | *Vernonia yellow vein Fujian betasatellite* | VYVFuB | JF733779 | *Vernonia yellow vein Fujian virus* | China | *Vernonia cinerea* | Asteraceae |
| 66 | *Vernonia crinkle betasatellite* | VCrB | KX831134 | *Vernonia crinkle virus* | Uganda | *Vernonia amygdalina* | Asteraceae |
